# An improved Bayesian network method for reconstructing gene regulatory network based on candidate auto selection

**DOI:** 10.1186/s12864-017-4228-y

**Published:** 2017-11-17

**Authors:** Linlin Xing, Maozu Guo, Xiaoyan Liu, Chunyu Wang, Lei Wang, Yin Zhang

**Affiliations:** 1 School of Computer Science and Technology, Harbin Institute of Technology, Harbin, China; 2 School of Electrical and Information Engineering, Beijing University of Civil Engineering and Architecture, Beijing, China; 3 Institute of Health Service and Medical Information, Academy of Military Medical Sciences, Beijing, China

**Keywords:** Gene regulatory networks, Bayesian network, Candidate auto selection, Breakpoint detection, Search space reduction

## Abstract

**Background:**

The reconstruction of gene regulatory network (GRN) from gene expression data can discover regulatory relationships among genes and gain deep insights into the complicated regulation mechanism of life. However, it is still a great challenge in systems biology and bioinformatics. During the past years, numerous computational approaches have been developed for this goal, and Bayesian network (BN) methods draw most of attention among these methods because of its inherent probability characteristics. However, Bayesian network methods are time consuming and cannot handle large-scale networks due to their high computational complexity, while the mutual information-based methods are highly effective but directionless and have a high false-positive rate.

**Results:**

To solve these problems, we propose a Candidate Auto Selection algorithm (CAS) based on mutual information and breakpoint detection to restrict the search space in order to accelerate the learning process of Bayesian network. First, the proposed CAS algorithm automatically selects the neighbor candidates of each node before searching the best structure of GRN. Then based on CAS algorithm, we propose a globally optimal greedy search method (CAS + G), which focuses on finding the highest rated network structure, and a local learning method (CAS + L), which focuses on faster learning the structure with little loss of quality.

**Conclusion:**

Results show that the proposed CAS algorithm can effectively reduce the search space of Bayesian networks through identifying the neighbor candidates of each node. In our experiments, the CAS + G method outperforms the state-of-the-art method on simulation data for inferring GRNs, and the CAS + L method is significantly faster than the state-of-the-art method with little loss of accuracy. Hence, the CAS based methods effectively decrease the computational complexity of Bayesian network and are more suitable for GRN inference.

## Background

Life activities are regulated through complex interconnections of genes and their products [1]. These interactions between genes form so-called gene regulatory networks (GRNs) in living cells. Inferring Gene regulatory networks (GRNs), also known as reverse engineering, is a critical problem in computational biology [2–4]. The advent of high throughput technologies has provided such an opportunity to biologists and bioinformatics researchers so that they can collect large amount of omics data that can quantify the activities of genes or their products. GRNs that constructed from gene expression data reflect the interactions of the regulatory elements in biological systems, such as genes and proteins [5–7], and the structure of GRN reveals the inner complex mechanism in adaptability to the environment and the growth and development of organisms [1, 8]. So enthusiasm for inferring GRN has continued unabated for years.

The availability of transcriptome data have been immensely improved by high throughput technologies such as DNA microarrays in recent years. This has led to the fast development of computational approaches for the reconstruction of GRN [9]. In computing complexity aspect, there are also various degrees of flexibility for modeling GRNs that range from complex differential equation method [10] to simple methods based on correlation coefficients [11]. Each model has its own special feature: pairwise or systematic, linearity or nonlinearity, etc.

The pairwise methods, which are relatively simple way, compute the correlation coefficients of genes and then set different threshold to construct GRNs [12, 13]. Commonly used methods to calculate correlations include Pearson Correlation Coefficient (PCC), mutual information (MI) [14], Granger causality [15, 16], etc. Most pairwise methods are low complexity, fast computing speed and adapting to large data set. Nevertheless, most of pairwise methods cannot identify the directions of regulatory interactions and cannot identify casual connections on system level. In addition, pairwise methods suffer from false positive/negative problems due to the simplicity of model and uncertainty of parameters.

Rather than the pairwise method, systematic approaches try to model the GRN from a holistic perspective. There are mainly three types of mathematical model in systematic approaches: Boolean network method [17–20], Bayesian Network method [21–24] and differential equation method [25–27]. These systematic methods can provide the researchers a deeper understanding of the regulatory mechanism at network level and can also identify the directions of regulations in the network. However, the problem of computational complexity makes them difficult to handle large-scale networks. With gradually increasing of computing complexity, the data size they can process rapidly goes down. Boolean network, which was first introduced by Kauffman [28], uses a set of Boolean variables and Boolean functions to describe gene-gene interactions. Probabilistic Boolean network, first introduced by Shmulevich et al., is a stochastic extension of Boolean network that integrates rule-based dependencies between variables [29]. Obviously, these crude simplifications of genes and their interactions cannot reflect the genetic reality. Differential equation method uses a set of differential equations to directly describe dynamic changes of the mRNA content in a precise manner. Obviously, the differential equation method can capture more details about the regulation relationships, but we could not bear such a high degree of computational complexity in most cases. Bayesian Network methods is in the middle of all the methods in complexity and scale. Bayesian Network is a probabilistic graphical model and tries to find a directed acyclic graph (DAG) that fits the expression data reasonably. Among all the models, BN is always a concern, because of its inherent probabilistic nature. In this paper, we focus on developing systematic methods based on Bayesian network to construct GRNs with higher accuracy and better scalability.

Yet, the Bayesian network method has some limitations. The reconstruction of GRN based on Bayesian network is NP-hard with respect to the number of genes, so the exact network structure can be learned only for relatively small datasets [30]. For large-scale networks, some variants of heuristic approaches are applied [31]. Due to the decomposable scoring function and some reasonable assumptions, the score-search framework for learning network structures are efficient [32]. However, heuristic methods do not guarantee the globally optimal network structure. Furthermore, most of the time is wasted on examining unreasonable candidates due to the scale and sparsity of biological networks. Hence, many researchers have devoted themselves to accelerating the learning process through reducing the search space [33]. Sparse candidate [21], maximum number of parents limitation [34] (also called maxP technique) and Max-Min Hill-Climbing (MMHC) [35] are typical methods for speeding up the structure learning.

The sparse candidate method is an iterative optimal algorithm by combining two steps: restricting the parents of each variable to a small subset of candidates and then searching for a network structure that satisfies these constraints. The learned network will improve the quality of candidates in the next iteration. The optimal GRN structure is learned by taking this iteration strategy. The maxP technique further simplify this idea by directly limiting the maximum indegree of each node. In the learning process, the parents of a node no longer increase until the indegree threshold is reached. The MMHC algorithm uses a more reasonable way to learn the candidates. It learns the neighbors of each node by using a local neighbor discovery algorithm called Max-Min Parents and Children (MMPC) [35].

**Fig. 1:**
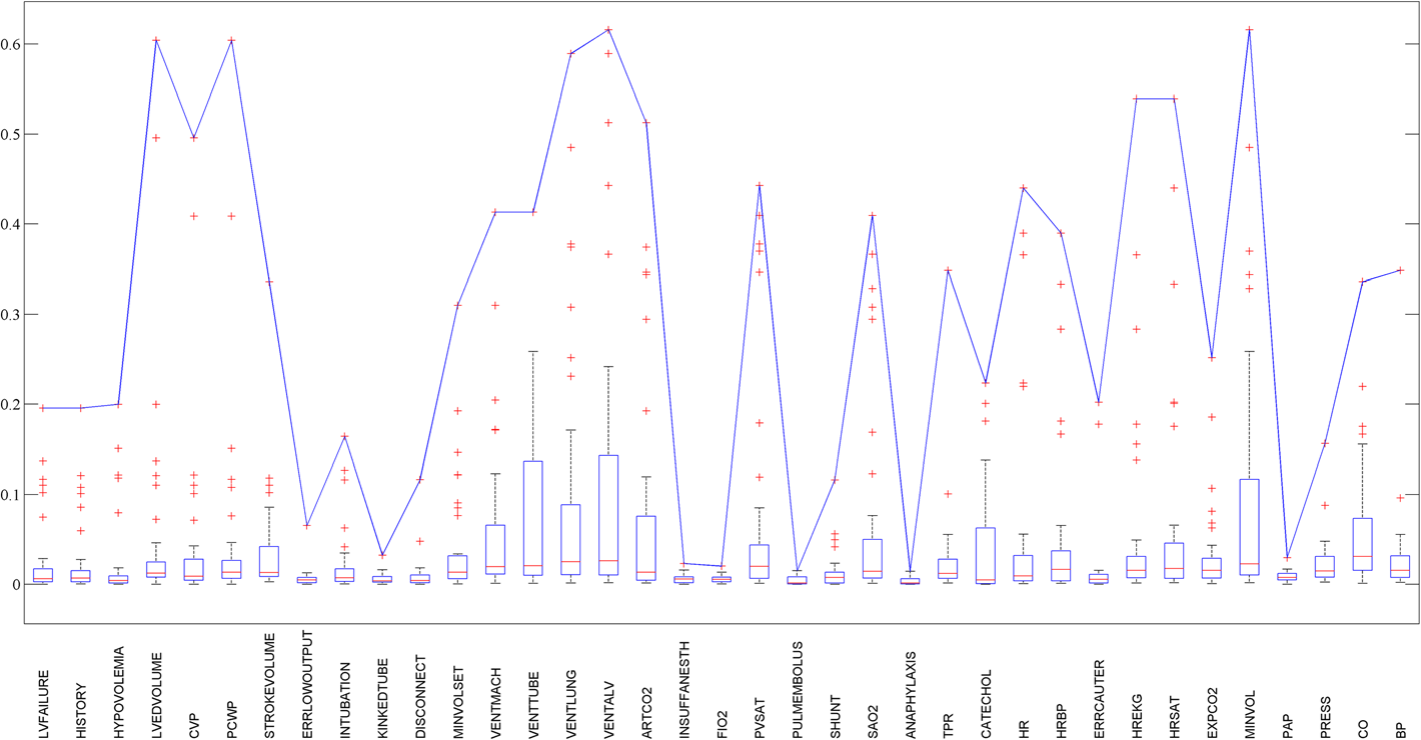
Mutual information distribution of each node in alarm network

Commonly, an important tuning parameter *k* is needed to indicate the size of candidate set or the maximum number of parents in sparse candidate or maxP algorithms. However, we are still far away from understanding the complex regulation mechanism of biological networks, so that we know rather little about GRNs to guide the selection of parameter *k*, even for model organisms [36, 37]. According to statistics in Table 1, degree distributions of known biological networks have obvious differences. This critical problem illustrates that the estimation of degree distribution using known networks is unreasonable. Therefore, no reliable estimates exist for parameter *k*. The arbitrary selection of parameter *k* ignores the complexity of biological networks and compulsorily cut the search space in a rigid manner. How about using unified correlation threshold to select candidates? Figure 1 shows the MI distribution of each node in alarm network. As we can see in Fig. 1, the distribution varies significantly at different node. Hence, the selection of candidates is also unreasonable through a unified correlation threshold, e.g. MI.

**Table 1:** indegree distribution of different network

–	% of total gene	
Indegree	E.coli	Yeast
1	5.1757	0.698
2	37.4441	35.6902
3	22.9393	24.454
4	13.8658	14.5913
>4	8.754	7.9261

MMHC’s heuristic heavily depends on the accuracy of conditional independence estimation. In MMPC step, the number of samples grows exponentially to the size of the conditioning set for accurately estimating the conditional independence. It is impossible to obtain so much data in a biological sense. So you can see that the situation, typically known as “large p, small n” problem, greatly limits the use of MMPC method for biological network reconstruction. Moreover, users also need to set a *p* value as the threshold of independence. Hence, the MMPC algorithm does not work well on small dataset, and leads to a huge amount of false positives.

Based on the analysis above, we can draw the following conclusions: a) Screening of candidates using global parameters is unreasonable and unrealistic. These methods cannot reduce searching time for examining unreasonable candidates and cannot solve the dependency on tuning parameter. In addition, we know little to guide the selection of the tuning parameters. b) Overly complex model is not suitable for GRN inference due to the limitation of biological data.

As is known to all, MI draws much attention in biological data analysis because of its ability to measure the non-linear relationships, which are common in biology. Previous studies has discussed and compared the advantages and power of using MI in measuring non-linear relations [38, 39]. And according to Frenzel’s work [40], MI can differentiate between direct and indirect interactions to some extent. Thus far, many researchers have used the MI measure in biological data analysis and network reconstruction, and get some achievements [41–43]. Some pairwise methods, such as CMI, also use an improved MI method as measurements of independence [44].

In this work, we proposed a novel candidate selection algorithm based on mutual information and the concept of breakpoint detection, named CAS (candidate auto selection). The CAS algorithm is designed to reduce the search space of structure learning and get rid of an unwanted dependency on tuning parameters. Firstly, the CAS algorithm utilizes the capability of MI to measure the non-linear regulatory interactions. Then, the candidate selection problem is formalized as a hypothesis test problem by using breakpoint detection. More importantly, this algorithm is a polynomial-time approach and do not depend on turning parameters. Further, based on the CAS algorithm, a globally optimal greedy algorithm (CAS + G) and a local learning algorithm (CAS + L) are also proposed for reconstruction of GRN. The proposed CAS + G algorithm aims at finding the optimal BN structure from data in the restricted search space. Meanwhile, the CAS + L algorithm which learning the structure in a local way pays more attention to the learning rate at little expense of quality. To evaluate the proposed methods, they are compared with a state-of-the-art method on different datasets.

## Methods

**Fig. 2:**
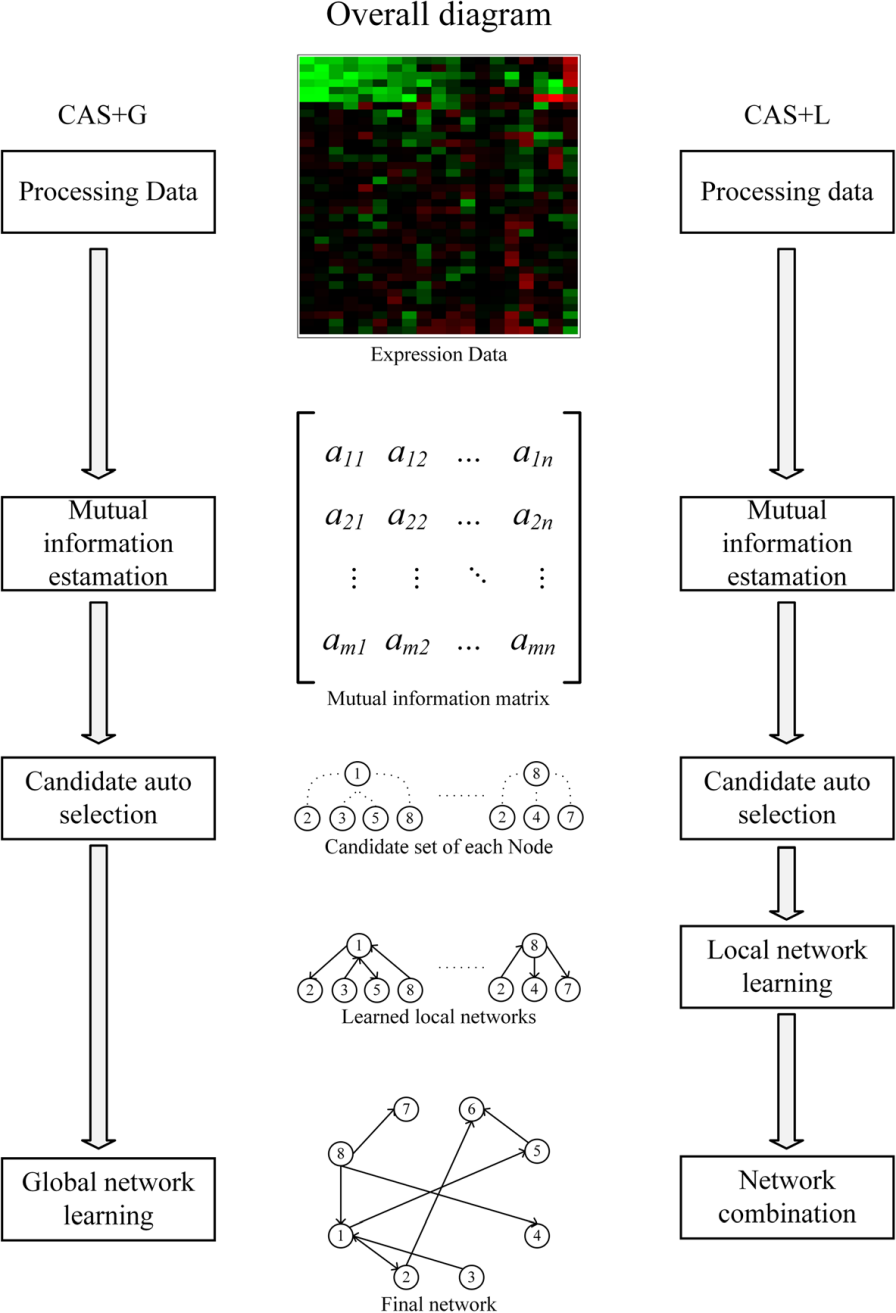
Overall diagram of our method. 1) process the expressing data, 2)estimate mutual information of each pair of genes, 3) construct candidate set for all nodes using CAS algorithm 4) learn sub-networks on each node for CAS + L (right column), 5) combine all sub-networks into the final network for CAS + L(right column), or directly learn the final network for CAS + G(left column)

This section consists of three parts: a) Candidate auto selection algorithm based on mutual information and breakpoint detection. b) Local learning algorithm (CAS + L) for reconstruction of the GRN. c) Globally-optimal greedy algorithm (CAS + G) for reconstruction of GRN. The overall diagram of aforementioned methods is shown in Fig. 2, the process on the left side is CAS + G and the process on the right side is CAS + L.

### A. Candidate auto selection (CAS) algorithm based on mutual information and breakpoint detection

Usually, MI is used as a metric of the correlation between two variables. Here we choose MI as a correlation measure of genes, which has been widely used to construct GRN from gene expression data due to its capability of capturing the non-linear relationships between genes as mentioned above. However, without considering the other variables, MI tends to overestimate the regulation strengths between genes (i.e., false positive problem). High MI value indicates that there may be a close relationship between the variables (genes) X and Y, while low MI value implies their independence.

MI of two discrete variables X and Y is defined as in (1):

1 $$ MI\left(X,Y\right)=-\sum \limits_{x\in X,y\in Y}p\left(x,y\right)\mathit{\log}\frac{p\left(x,y\right)}{p(x)p(y)}=H(X)+H(Y)-H\left(X,Y\right) $$

where *p*(*x*, *y*) is the joint probability of *X* and *Y* under specific value x, y, and *p*(*x*), *p*(*y*) are the marginal probability; *H*(*X*), *H*(*Y*) are entropies of X,Y; *H*(*X*, *Y*) are joint entropy of *X* and *Y*.

**Fig. 3:**
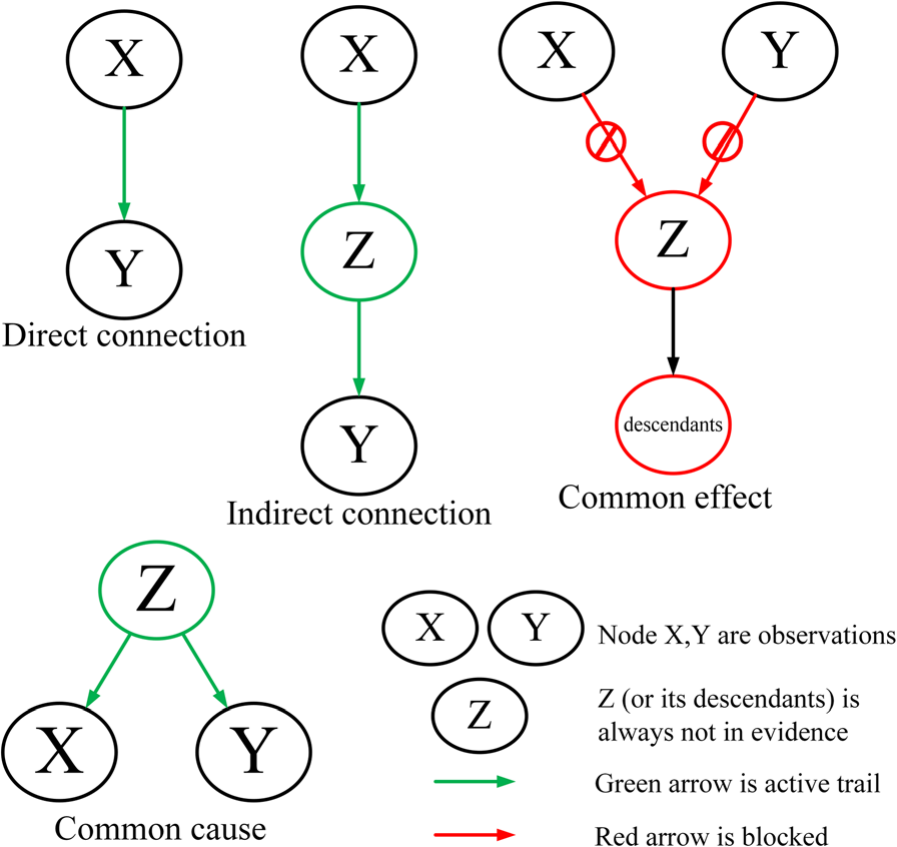
Possible relationships between X and Y

Obviously, in a real case of inferring GRN, node Z or its descendants are always not in evidence. Hence, we show all the possible relationships of node *X* and *Y* in the case of no other observations, as shown in Fig. 3: Node X is closely related to Y in the case of direct connection and common cause, the correlation will decrease with distance in the case of indirect connection, and Node X and Y are independent with each other in the case of common effect (also known as V-structure). In biological networks, the correlations between a gene and its regulators or targets are closer than that between this gene and irrelevant genes. That is, the correlations between a gene and the genes in common effect branch is different corresponding to Fig. 3.

With the above analysis and mentioned research [32, 44], we can conclude that the MI distribution of highly correlated nodes is different from the distribution of uncorrelated nodes. According to this conclusion, a mutual information and breakpoint detection based method is proposed in this paper aiming at exactly selecting the candidates. This method achieves the goal of reducing search space by cutting off all the unrelated nodes in common effect branch.

To describe the whole method, we start by calculating the potential neighbors of one target node. The process of calculating the potential neighbors of one target node is as follows. The input consists of the data *D* on the node set *V *of size *n* and a target node *g*_*i*_ ∈ *V*. The output is candidate set *C*_*i*_ for node *g*_*i*_, which consists of the potential neighbors of *g*_*i*_. In the first step, all the MIs between node *g*_*i*_ and other nodes are computed and stored in vector $\mathcal{X}$. These MIs are summarized based on ascending order of values. At this time, Vector $\mathcal{X}$ of size n-1 stores all the MIs between node *g*_*i*_ and other nodes in ascending order. Then suppose there is a position (breakpoint) in vector $\mathcal{X}$ that divides the nodes into two parts: related nodes and unrelated nodes. The task of breakpoint detection can be formalized as a hypothesis-testing problem, which can be solved using maximum likelihood method. So this breakpoint can be found by constructing a statistic and locating the maximum. The nodes on the left side of the breakpoint, whose MI value is smaller than the MI value of the breakpoint, are identified as unrelated and discard. The nodes on the right side of the breakpoint with bigger MI values are added to candidate set *C*_*i*_. Then nodes in set *V* are processed one by one.

Now, the key question is how to construct the statistics and do hypothesis testing. As mentioned above, given node *g*_*i*_’s MI vector $\mathcal{X}$, the goal is to determine whether a significant breakpoint exists or not. The null hypothesis and the alternative hypothesis are stated as follows:

H0: Null hypothesis -- no breakpoint exists.

H1: alternative hypothesis-- one significant breakpoint exists.

Then we construct statistic Q to decide whether or not the null hypothesis should be rejected in hypothesis testing. The incoming data is the MI vector $\mathcal{X}=\left\{{x}_1^{\hbox{'}},{x}_2^{\hbox{'}},\dots, {x}_m^{\hbox{'}}\right\},m=n-1$. Based on the prior analysis, we suppose that the MI of node *g*_*i*_ with related nodes and node *g*_*i*_ with unrelated nodes are coming from different distributions. For a model with a breakpoint at *k* ∈ [1, *m*]k ∈ [1, m], the maximum likelihood is defined as in (2)

2 $$ ML(k)=\log \left(p\left({\mathcal{X}}_{1:k},{\theta}_1\right)\right)+\log \left(p\left({\mathcal{X}}_{k+1:n},{\theta}_2\right)\right) $$

Where *p*(*X*| *θ*) in (2) is the probability density function, *θ*_1_, *θ*_2_ is the corresponding parameters of each distribution. Now the testing statistic *Q* can be defined as (3):

3 $$ Q=2\left[ ML(k)-\log \left(p\left({\mathcal{X}}_{1:n}|\theta \right)\right)\right] $$

*p*(*X*_1 : *n*_| *θ*) in (3) is the probability of the null hypothesis.

Usually, we need to choose a constant value *c* as the threshold. If *Q* > *c*, then we reject the null hypothesis. At this point, all the position *k* satisfying *Q* > *c* are breakpoints which dividing the vector into two different parts. Obviously, here *c* is underdetermined. However, for the goal of finding the best candidates, the maximum of *Q* can be considered as the criterion to determine the best breakpoint, as in (4).

4 $$ k= argmax(Q) $$

The nodes on the left side of the breakpoint *k* are unrelated nodes in common effect branch. Then all the nodes corresponding to MI on the right side of the breakpoint *k* are identified as the candidates.

At this time, let us solve the problem of how to calculate the probability. That is to say, how to make reasonable assumptions about the MI distributions of two types of nodes. If two nodes (*g*_*i*_ and *g*_*j*_) are independent in the case of common effect, that is, knowing *g*_*i*_ does not give any information about *g*_*j*_ and vice versa, so their mutual information is zero by definition. However, due to the limit of sample size, the computed MI of these nodes are statistical noise, which can be modeled by a normal distribution. In other words, the MI distribution of unrelated nodes is a normal distribution with parameter *θ*_1_ = (*μ*_1_, *σ*_1_). Here, *μ*_1_ is the mean or expectation of the distribution. The parameter *σ*_1_ is its standard deviation. As we know, the normal distribution is often used to represent real-valued random variables whose distributions are not known. Therefore, it is also a reasonable assumption that the MI of related nodes is also normally distributed. Obviously, it has a different parameter *θ*_2_ = (*μ*_2_, *σ*_2_).

The full algorithm is as follows:
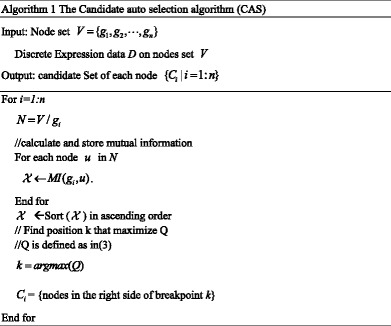


In algorithm CAS, the while loop is executed *n* times. The time requirement inside the loop is dominated by the procedure of finding breakpoint (the position k) to maximize the statistic *Q*. For finding the breakpoint of a specified node, we must go through all the possible locations of the MI vector. In this process, the posterior probability is calculated at most *n* times for each possible position. So the time complexity is *O*(*n* ^2^). Thus, the total time requirement of the algorithm is *O*(*n* ^3^).

### B. Local learning algorithm CAS + L for reconstruction of GRN

Local learning is a common idea in network structure learning problem. Based on the proposed CAS algorithm above, we can obtain the neighbors of each node exactly. Thus, local learning is a good solution for inferring the network structure in this case. Hence, we present a local learning method based on the CAS algorithm for inferring GRNs. To describe the whole idea of this local learning algorithm, we start with learning the local structure of a specified node. In the first step, we compute the candidate set *C*(*v*) of specified node *v* by using CAS algorithm. Then construct potential edge set *E*(*v*) of node *v*: *E*(*v*) = {(*v*, *u*), (*u*, *v*); *u* ∈ *C*(*v*)}. At last, a typical score-search framework is applied to finding the best local structure *G*(*v*) of node *v* on the search space defined by *E*(*v*). The high-level pseudocode of the full algorithm is as follows:
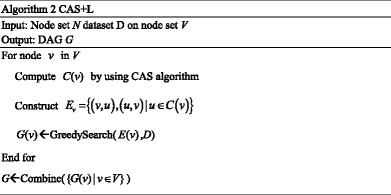


To combine all the subgraphs in a simple way, we accept the edge one by one, which do not introduce cycles. By analyzing this algorithm, we can find that this algorithm can be parallelized easily.

Given a node set *V* of size *n*, suppose that *k* candidates are selected for a node *v*. That means the algorithm should only check *k* possible candidates on one iteration. To learn the local structure of node *v*, 2 *k* candidate edges should be examined, so the search space complexity is *O*(2^2*k*^). The total time for learning the structure is *O*(*n* × 2^2*k*^). That is, the boundary of time is restricted by the size of candidate set. As we know, structure sparsity is one of the important properties of biological networks. At this point, we can suppose that the mean number of candidates is far smaller than *n*, e.g. *k* = log(*n*), then the space complexity is *O*(*n* × 2^2 log *n*^).

### C. Globally-optimal greedy algorithm (CAS + G) for reconstruction of GRN

The local learning algorithm is a quick solution, but does not guarantee a global optimum. For getting a global optimal solution, a global optimal algorithm based on the CAS method (CAS + G) is also proposed in this paper. The difference between the local method and the global method is that the CAS + G algorithm learning the structure as a whole in the restricted search space generated by CAS rather than learning substructures of each node.
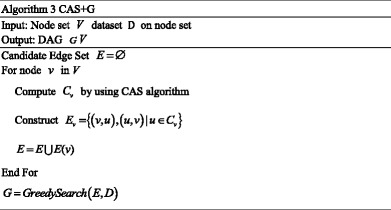


The greedy search procedure adds the highest rated edge at each iteration with the hope of finding a global optimal GRN structure. Moreover, the algorithm only needs to examine the limited edge set at each iteration. Let us suppose that the average size of candidate set is *k*. The search space complexity is *O*(2^*nk*^). At this point, we also suppose that the mean number of candidates is far smaller than *n* according to the sparsity of the structure, e.g. *k* = *n*/4 is a small constant *c*, then the space complexity is *O*(2^*n* × *n*/4^).

## Result and discussion

### Used networks and datasets for evaluation

In order to evaluate the CAS algorithm presented in this article, experiments are carried out on two types of networks: a. known Bayesian networks, b. Dream challenge networks. Known Bayesian networks are constructed by experts, and all the parameters are known. These networks are insurance network (27 nodes, 52 arcs), alarm network (37 nodes, 46 arcs), Barley network (48 nodes, 84 arcs), Hailfinder network (56 nodes, 66 arcs). Five datasets of different sizes (*n* = 50, 100, 200, 500, 1000) are sampled from each network. Ten 100-gene networks are collected from DREAM3 and DREAM4 challenge. The corresponding simulation data (210 samples) for these in-silico networks are generated by GNW software [45] and then converted into discrete data by K-Means discretization algorithm.

### Results of CAS algorithm

In this section, the effectiveness of the CAS algorithm is illustrated and compared with MMPC algorithm. The aim of the CAS and MMPC algorithm is to identify the neighbors of each node in as few candidates as possible. So, in classification point, the true positive (TP) is defined as neighbor nodes correctly identified as candidates, and the recall in this context is defined as the number of true positives divided by the total number of the neighbors, as in (5).

5 $$ recall=\frac{TP}{TP+ FN} $$

False negative (FN) are neighbor nodes incorrectly identified as unrelated nodes. Therefore, TP + FN equals to the number of true neighbors. In the following test, the recall and the average number of candidates are the most important indicators.

We studied the influence of the network size, network type and the dataset size to validate the effectiveness of the CAS method on the aforementioned networks. The alarm network is analyzed in detail.

**Fig. 4:**
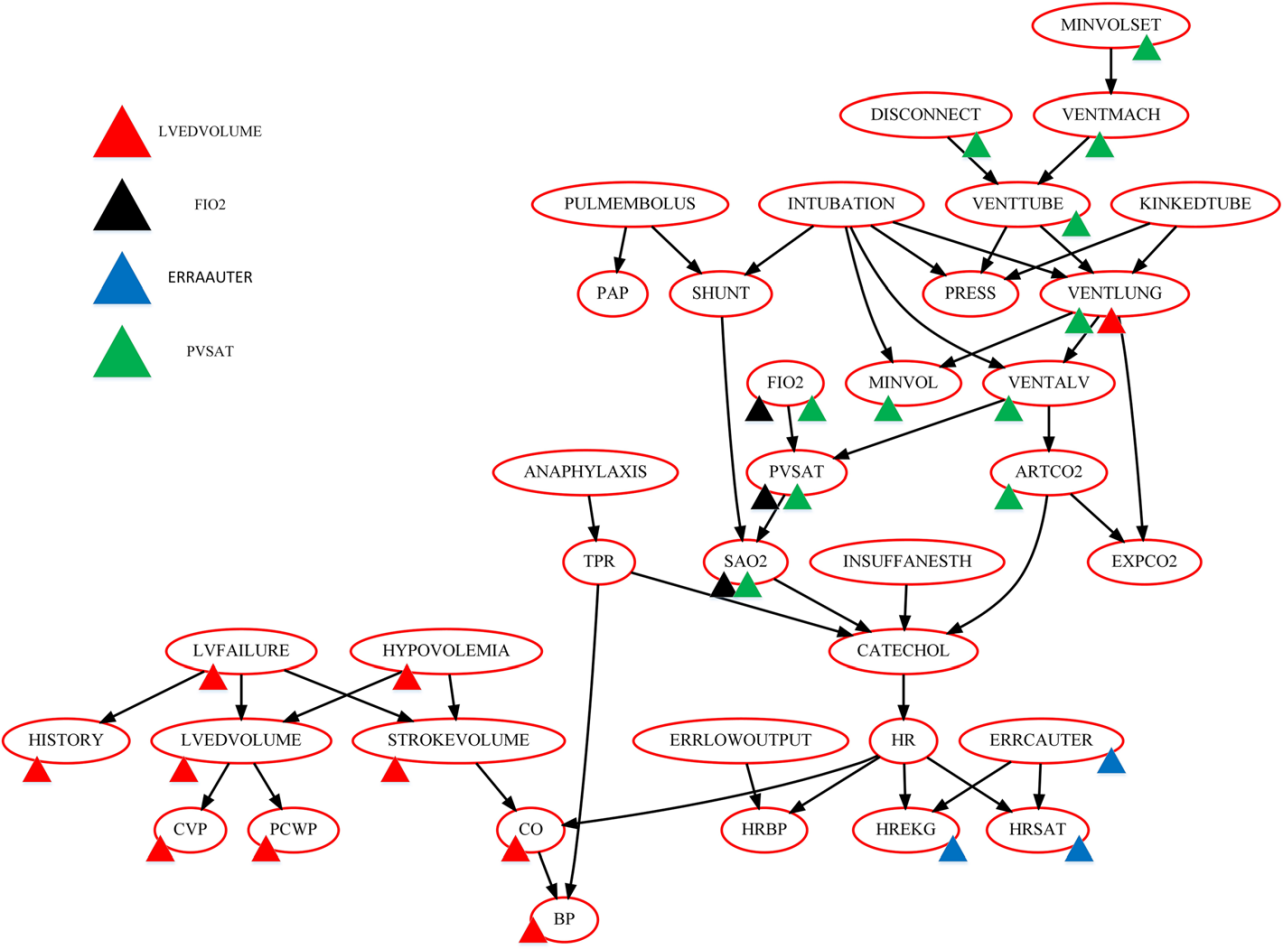
Identification result of CAS algorithm on alarm network. Red triangle indicates the candidates of LVEDVOLUME identified by CAS algorithm. Likewise, the black triangle and blue triangle green triangle correspond to the candidates of FIO2, ERRCAUTER, PVAST, respectively

We mark the identification result of four representative nodes on the true network to illustrate the detail result of CAS algorithm. As shown in Fig. 4, triangles with four colors indicate four different nodes in alarm network: red, black, blue and green corresponding to LVEDVOLUME, FIO2, ERRCAUTER, PVSAT, respectively. LVEDVLOUME (indicated by red triangles) has two parent nodes and two sibling nodes. Figure 4 shows that the CAS algorithm identified 9 candidates, and these 9 candidates completely cover all 4 true neighbors. Meanwhile, the candidates except VENTLUNG are closely related to LVEDVOLUME as mentioned in Fig. 3. For node FIO2, the candidates identified by CAS contains its child node. In addition, the common effect branch of FIO2 (the parents of PVSAT) is correctly cut off by CAS algorithm. For node ERRCAUTER, when HREKG and HRSAT are not given, HR and ERRCAUTER are independent in the common effect case. Hence, as mentioned in the method section, its child nodes (HREKG, HRSAT) are correctly identified by the CAS algorithm and the nodes in the common effect branch (all ancestors and descendants of node HR) are discarded. At last, we analyze another representative node PVSAT, which has a complicated patrilineal family. In this situation, the CAS algorithm also identified its parents and child correctly, and discard the nodes in common effect branch. We can see that the candidate set cover more ancestors rather than descendants. This phenomenon illustrates that node PVSAT is more affected by its ancestor and is not closely related to its descendants.

By analyzing the results of CAS algorithm on all nodes of alarm network, we can conclude that the CAS algorithm can effectively discard the unrelated nodes in common effect branch and can identify most of the true candidates correctly in most cases. In addition, the identification rates increase simultaneously with the amount of data. Nevertheless, there are still a few nodes that cannot be identified correctly and these situations are not improving with the amount of data in our experiment. Further analysis indicates that the generated data cannot reflect the real probability distribution correctly due to its very small conditional probability in the conditional probability table. Hence, the generated data could not provide enough power to distinguish its neighbors and other nodes, such as INSUFFANESTH. For these nodes, more data is needed to confirm its correlation with others.

**Fig. 5:**
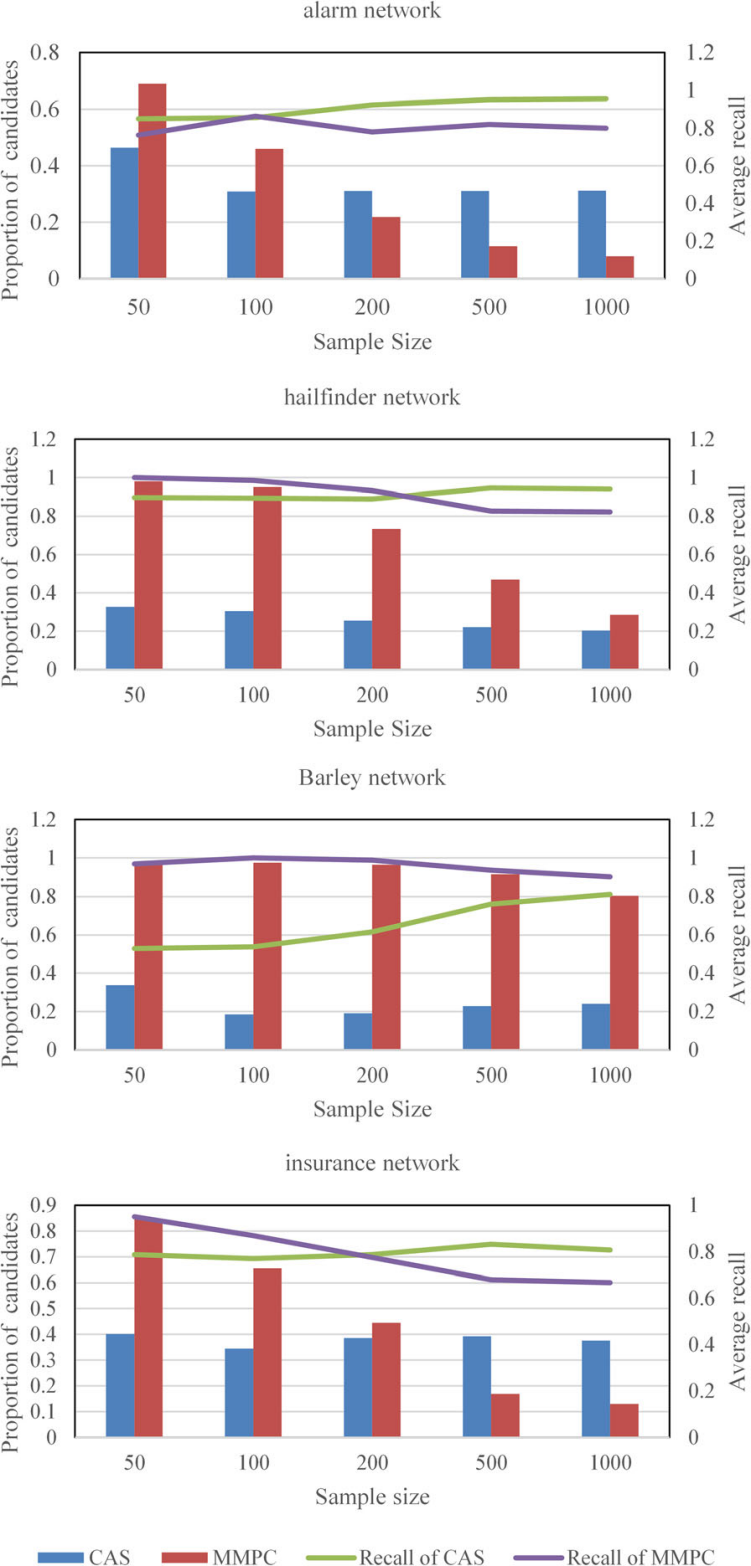
Average recall and the proportion of candidates on different networks. In this figure, subgraphs (from top to bottom) correspond to alarm network, Hailfinder network, Barley network, insurance network, respectively. Solid lines corresponding to secondary axis are changing trends of average recall

Figure 5 summarizes the trends of recall and the average number of candidates of CAS and MMPC on different networks when sample size gets larger. Based on the analysis of all these charts, the average recall of CAS (green line) increases with sample size, while the average number of candidates (blue bar) decreases. This situation illustrates that sufficient samples can improve the performance of CAS algorithm. Nevertheless, there are different situations in MMPC method. The average number of candidates decreases as samples increase in numbers (red bar), but the recall also declines to different extents (purple line).

As we can see from Fig. 5, when the sample size is small, the MMPC algorithm takes nearly all nodes as neighbors. This means that MMPC algorithm breaks down because of the lack of samples. Especially, it is more obvious in Barley network. That is mainly because multivariate variables in Barley network, so that the data is not enough for accurately estimating the conditional associations. In insurance network, the decrease of average recall is mainly because of the undue screening. Hence, the MMPC cannot provide adequate performance when the data is insufficient. However, the CAS algorithm has better filtering ability in most cases. For example, the CAS algorithm gives out less candidates than MMPC algorithm for nearly the same recall rate in alarm network and Hailfinder network, when the data size is 50. Even in barley network, the CAS algorithm still shows better performance than MMPC with the increase of samples. We can draw a conclusion that the CAS algorithm is more effective on small data sets. Thus, it is more suitable for GRN inference which is a typical “large p, small n” problem.

Moreover, to assess the effectiveness further, the evaluations are also carried out using the simulation data from DREAM challenge. Although the complexity of the biological networks and the imperfection of simulation data will impose some performance penalties, it remains effective to a much larger degree, which will be validated in the learning phase. The CAS algorithm can effectively reduce the search space through cutting off the common effect branch. Based on the above analysis, one can draw a conclusion that the CAS algorithm outperforms the MMPC algorithm, especially on small data sets.

### Evaluation of the CAS + G and CAS + L algorithm for inferring GRNs

The performance of structure learning was evaluated using the following measures: TP, FP, TN, FN, Precision, Accuracy, Recall, Specificity and F-score. Experimental comparisons of CAS based methods against MMHC on various sample sizes are carried out. A local learning algorithm named MMHC + L using MMPC as the candidate selection algorithm is also in comparison. For purpose of comparison, we choose original greedy search as benchmark method, which applies the score-search framework to find the optimal structure. The leading reason for the choice of greedy search is that there are no limits on the search space with the hope of finding a global optimum. Hence, the performance improvement is the ratio of the performance of a particular algorithm to the performance of the benchmark. Here BDeu score [46] is selected from other well-known scores. We examine all the networks described above, but only some are analyzed in detail limited by the space. Firstly, the alarm network and insurance network are analyzed and discussed.

**Fig. 6:**
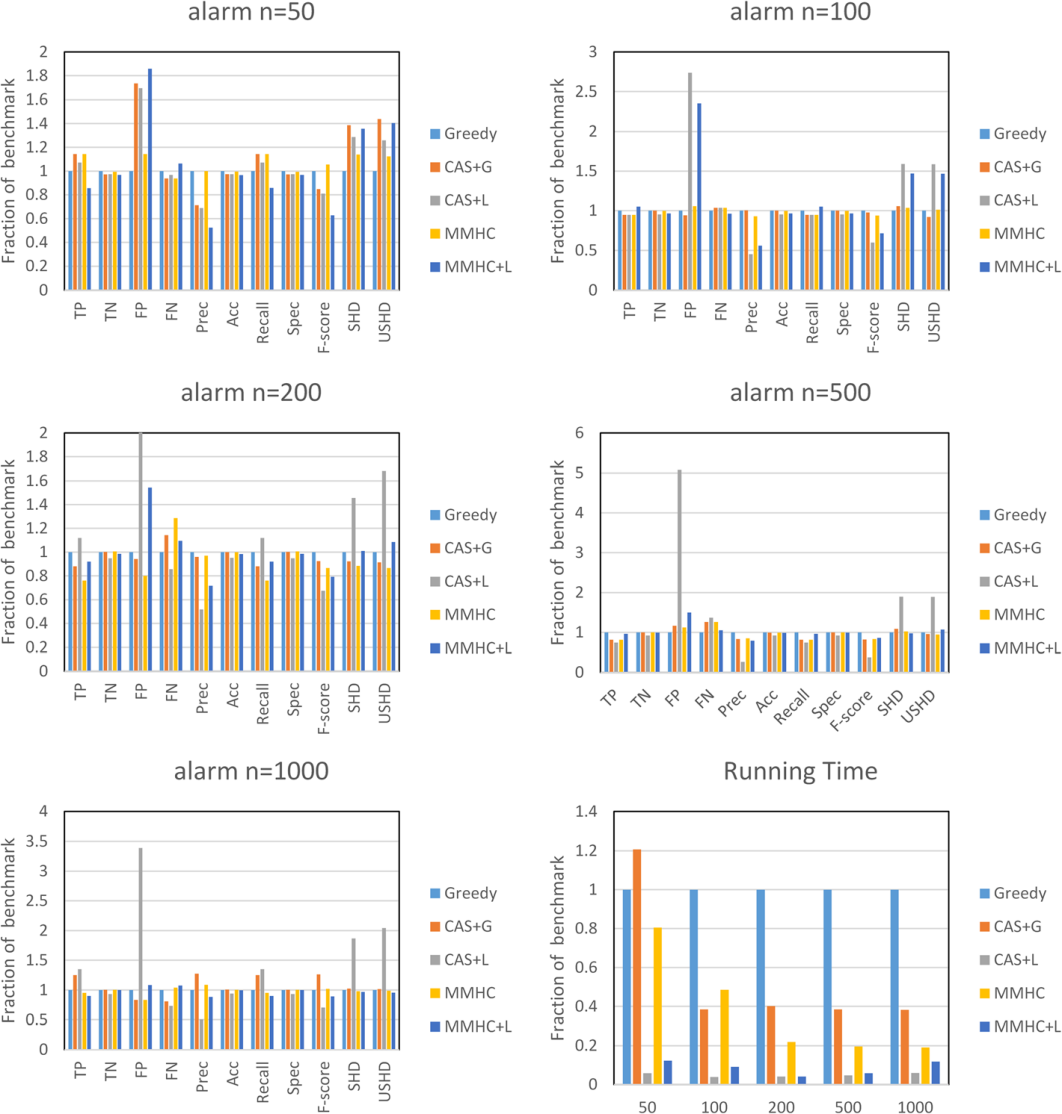
The performance improvement on alarm network using CAS + G algorithm and CAS + L algorithm compared with normal Greedy search as benchmark

**Fig. 7:**
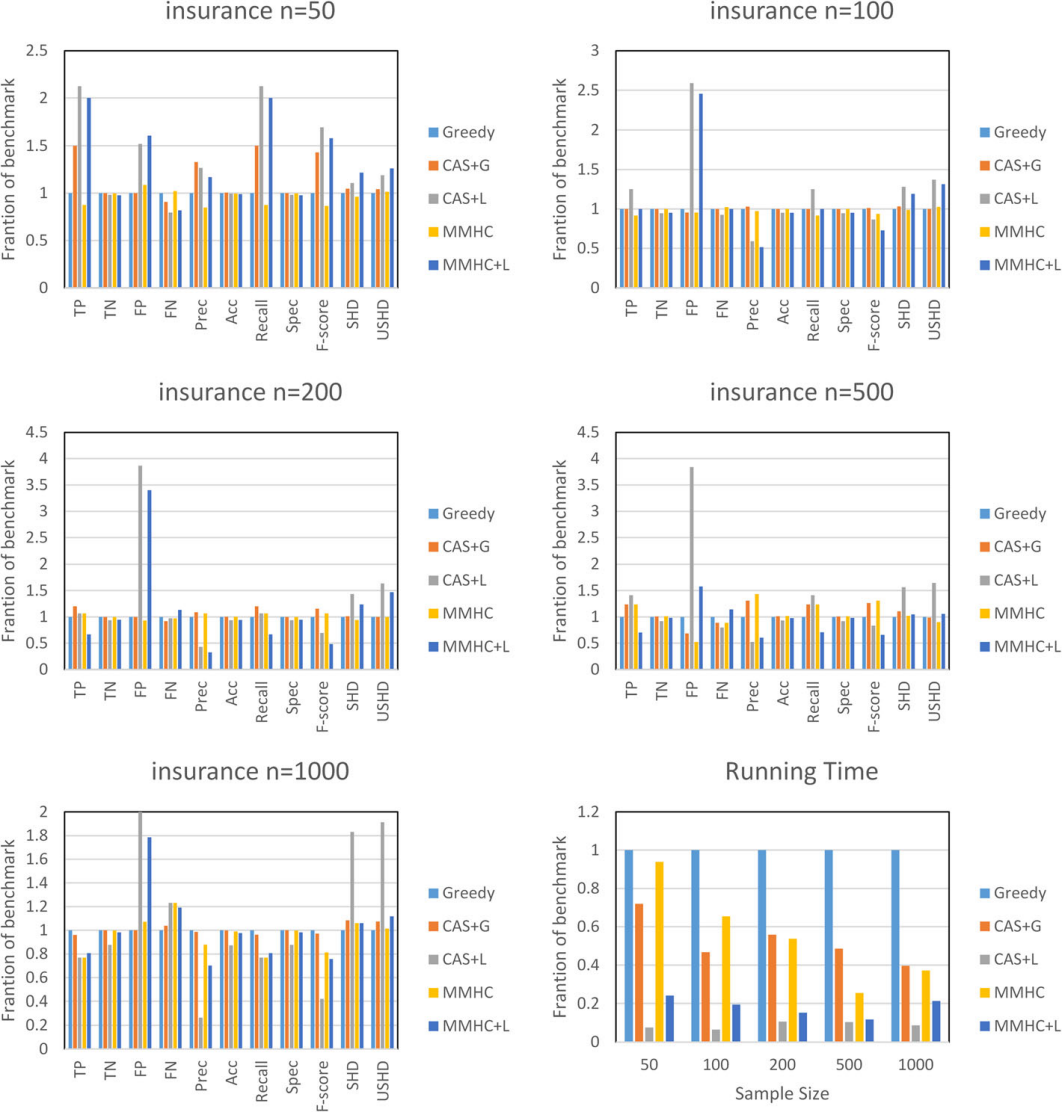
The performance improvement on insurance network using CAS + G algorithm and CAS + L algorithm compared with normal Greedy search as benchmark

Figures 6 and 7 show the comparison result on the alarm and insurance network in different sample size. The first five charts corresponding to five different sample sizes show comparison results under aforementioned measures. The sixth subgraph shows the runtime result with different sample sizes. When sample size is 50, the behavior of MMHC is nearly the same as greedy search according to the candidate selection results in Fig. 5. As you can see by comparing subgraphs 1 to 5, the CAS + G algorithm outperforms the MMHC algorithm with the increase of sample size, mainly because ample data can improve the performance. Especially in insurance network, the CAS based methods outperform MMPC based methods in all sample sized. It is not just about the learning phase, but also about the quality of candidate selection, which generates a more exact search space. When the sample size is larger to more accurately estimating the conditional independence, the performance of MMHC algorithm gets better quickly. Nevertheless, when the sample size is 1000, the recall of the MMPC algorithm is much lower than CAS according to Fig. 5. That is, MMPC overly reduces the search space and rejects many true neighbors. Finally, the proposed global optimal algorithm CAS + G is superior to the benchmark and better than MMHC when the sample size is 1000.

The sixth subgraph is the runtime results of different sample sizes. Firstly, we can see that the proposed CAS-based learning methods run more stable than MMPC-based methods and greedy search by taking into the number of candidates. In particular, CAS + L algorithm has an obvious advantage on runtime in both networks, especially in insurance network. The CAS algorithm does not need to enumerate all the conditional sets, so it is much faster than MMPC algorithm. Hence, the total time of CAS + L algorithm is less than the other methods. For the CAS + L algorithm, the main advantage is its speed without losing much of quality. Nevertheless, much more false positives are found because of fluctuations in the data and the simple combination phase. It may be solved by replacing the simple combination method with a heuristic phase.

In general, sufficient samples provide a performance boost because of the improvement of CAS algorithm and the score-search phase. There are still swings due to their heuristic nature, which easily trap into a non-optimal “local maximum”. According to the above analysis, the CAS algorithm is more suitable for small datasets.

Knowing the complexity of biological networks and the “large p, small n” situation, evaluation of the proposed methods should be made on biological datasets. Therefore, we carried out experiments on the simulation data of DREAM3 and DREAM4 challenge.

**Fig. 8:**
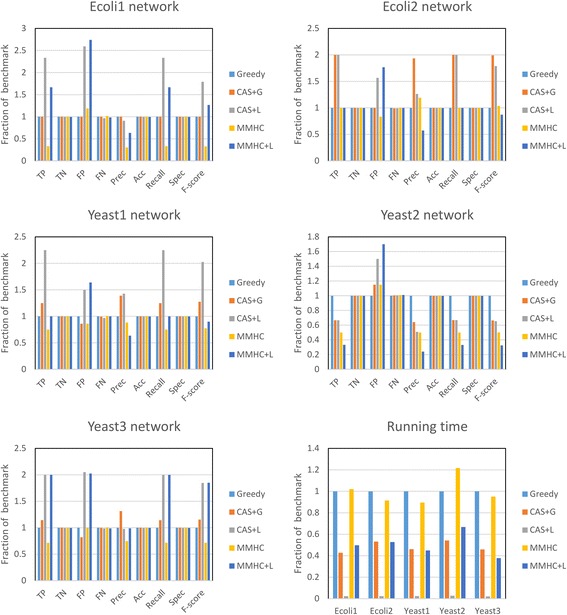
Comparison results of five 100-genes networks from DREAM3 challenge. The first five subgraphs show the comparison results of each network respectively. The last subgraph is the runtime comparison result

Figure 8 shows the comparison results of five 100-gene networks from DREAM3 challenge. As can be seen from Fig. 8, the CAS + G algorithm identified more true positives than MMHC method. Experimental results showed that CAS based method compared favorably against other approaches in the F-score and recall. As previously mentioned, the MMPC algorithm cannot accurately estimate the conditional association due to the lack of samples. Hence, the performance of MMHC algorithm is similar with Greedy Search. From the results of Yeast2 network, we can see that both algorithms trapped into local optimum due to the lack of samples. However, the performance of the CAS + G and CAS + L algorithms are better than MMHC algorithm, mainly because of the conciseness of CAS algorithm, which reduces data dependencies of the algorithms.

**Fig. 9:**
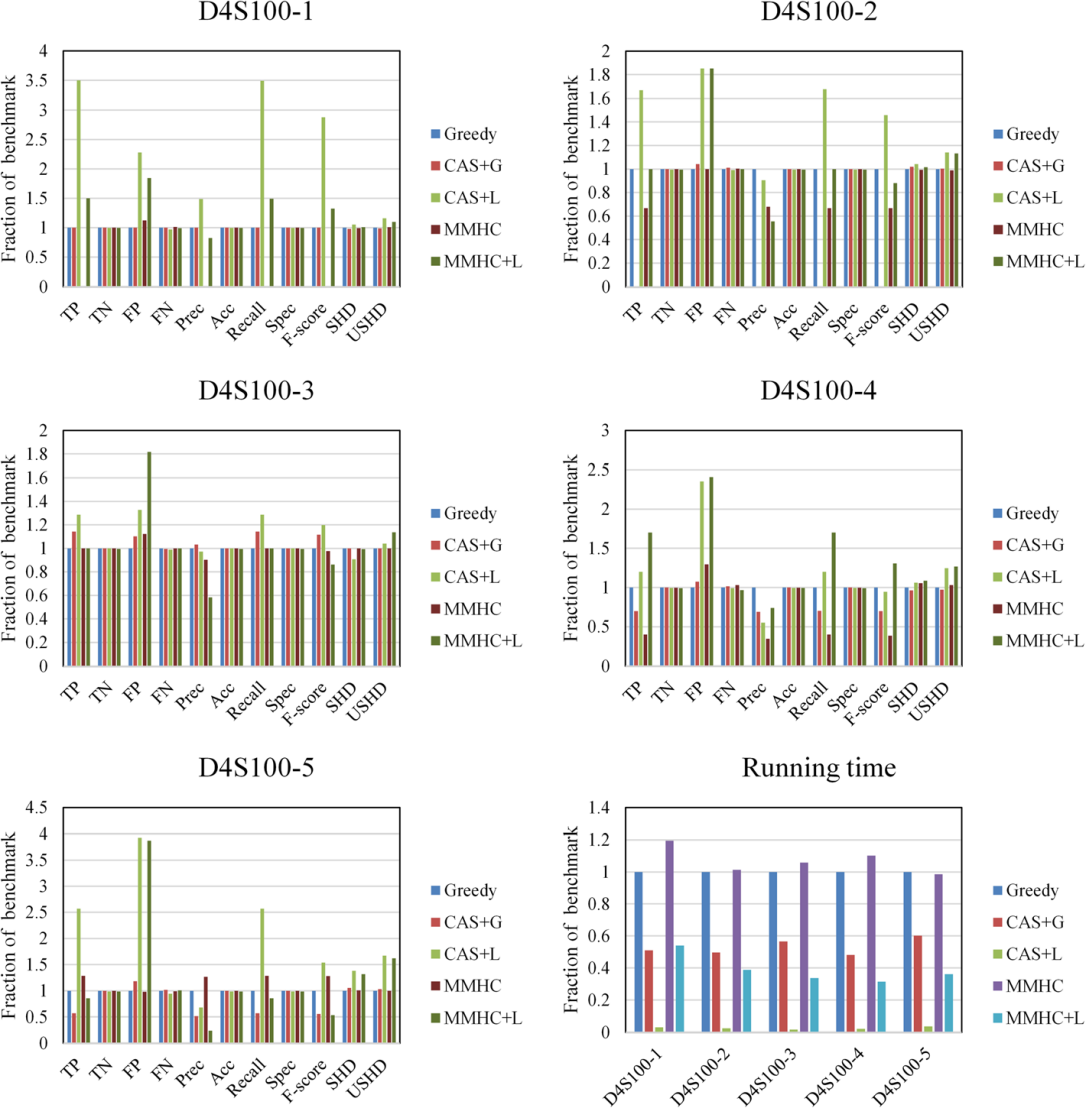
Comparison results of five 100-genes networks from DREAM4 challenge. The first five subgraphs show the comparison results of each network respectively. The last subgraph is the runtime comparison result

Figure 9 shows the comparison results of five 100-gene networks from DREAM4 challenge. As can be seen from Fig. 9, the performances are very similar to DREAM 3 networks overall. Experimental results showed that the CAS-based approaches outperform other approaches in the F-score and recall and identify more true positives than MMHC method on network 1, 3, 4. What we find once again here is that the performance of MMHC algorithm is similar to Greedy Search due to the limitation of sample size. However, the CAS + G algorithm identified less true positives on network 2 and 5. In addition, we even find the CAS + G algorithm failed on network 2. According to the analysis, we found that network 2 and 5 are more complex than others. This resulted in more performance degradation than the MMHC algorithm, which behaves exactly like Greedy search. Yet even so, MMHC algorithm has little advantage depending on SHD metric. Form the sixth subgraph, we find that the proposed CAS-based methods have an obvious advantage on runtime. Especially the CAS + L algorithm—it takes advantage of both the reduced search space and the locality. Nevertheless, much more false positives are found due to noise in the data and the oversimplified combination phase. We can draw a conclusion that the CAS + G algorithm has equal or better performance than the MMHC algorithm and greedy search for GRN reconstruction, but runs faster. Meanwhile, CAS + L algorithm runs significantly faster than all others.

The CAS algorithm has several advantages because of its conciseness and locality. First, there is no turning parameter to be determined. Erroneous estimation of turning parameter affects not only efficiency but also the quality of the reconstruction. This is very important for unknown situations of biological networks. The second one is its weak data dependencies, which makes it more suitable for GRN reconstruction. However, there are still limitations to consider, especially the assumption on the distribution of related nodes. The selection of Gaussian distribution is mostly based on experience and lack of a detailed study. In the future, we will study to make a more reasonable assumption. Another extension is to remove false positive candidates by using biological prior knowledge. In brief, we believe that the proposed CAS algorithm can identify the candidates effectively and improve the search process effectively. Furthermore, the global and local learning algorithms outperform the other methods on simulation data. It is clearly that the CAS based algorithms are very suitable for GRN reconstruction, which is always eager for data.

## Conclusion

In this work, we first proposed a novel method CAS to select candidates of each node automatically. This algorithm is designed to reduce the search space by restricting the neighbors of each node to a small candidate set. Firstly, MIs between nodes are calculated to reflect the independence. It is reasonable to assume that the distribution of the MIs of two types of nodes are different. That is to say, there is a breakpoint in the MI vector of each node to distinguish related nodes and unrelated nodes. Then, the breakpoint is located by hypothesis testing. So far, the candidates of each node are obtained. In the later learning phase, these candidates exactly restrict the search space. Hence, based on CAS algorithm, we propose a global optimal method (CAS + G), which focuses on finding the high-scoring network structure, and a local learning method (CAS + L), which focus on faster learning the structure with small loss of quality. At last, we validate the proposed algorithms on through experiments. Firstly, they are verified on known Bayesian networks. Then, the proposed methods are migrated to simulated biological data.

In candidate selection phase, the CAS algorithm correctly identifies the candidates of each node in polynomial time by discarding all nodes in common effect branch. The algorithm achieves relatively high performance on known Bayesian network datasets. However, it degrades on simulated data. Actually, it is not complicated to understand by considering the complexity of biological systems and the limitations of simulated data. The results show that CAS algorithm outperforms the MMPC algorithm. Especially, the CAS algorithm shows better performance on limited samples.

In structure learning phase, evaluations of CAS + G algorithm and CAS + L algorithm are carried out. The comparisons results show that the CAS + G method can learn the optimal structure and can avoid the local optimum to some extent benefited from the exactly restricted search space. Meanwhile, the CAS + L algorithm has obvious superiority in speed compared with other methods. Therefore, the proposed algorithms are effective and more suitable for GRN inference than MMHC algorithm.

Finally, the CAS algorithm is practical to reduce the search space, especially for limited samples, and provides enough flexibility to be extended in other fields. In the future, we would like to study the CAS algorithm in Markov Blanket view and consider a parallel implementation of the proposed algorithms.
